# Enhancing tumor deepfake detection in MRI scans using adversarial feature fusion ensembles

**DOI:** 10.1038/s41598-025-31231-7

**Published:** 2025-12-09

**Authors:** Aleem Ali, H. Anwar Basha, K. Thanuja, Puneet, Shashi Kant Gupta, SeongKi Kim

**Affiliations:** 1 Department of Computer Science & Engineering, UIE, Chandigarh University, Mohali, Punjab 140413 India; 2 Department of Computer Science and Engineering, Rajalakshmi Institute of Technology, Chembarambakkam, Tamil Nadu India; 3 School of Computer Science and Engineering, REVA University, Bangalore, Karnataka India; 4 Department of CSE, School of Engineering and Technology, CGC University, Mohali, Punjab 140307 India; 5 Lincoln University College, Petaling Jaya, Selangor Darul Ehsan 47301 Malaysia; 6 Adjunct Research Faculty, Centre for Research Impact & Outcome, Chitkara University Institute of Engineering and Technology. Chitkara University, Rajpura, Punjab 140401 India; 7 Institute of Well-Aging Medicare & CSU G-LAMP Project Group, Chosun University, Gwangju, 61452 Republic of Korea; 8 Department of Computer Engineering, Chosun University, Gwangju, 61452 Korea

**Keywords:** Adversarial training, Deepfake, Ensemble learning, Medical deepfakes, Tumor detection, Cancer, Computational biology and bioinformatics, Engineering, Health care, Mathematics and computing, Medical research

## Abstract

The proliferation of AI-generated medical deepfakes, such as tumor insertions or removals in diagnostic scans, threatens patient safety and healthcare integrity. Existing detection methods often lack robustness against adversarial attacks or fail to integrate multimodal feature representations. To address these gaps, we propose AFFETDS (Adversarial Feature Fusion Enhanced Tumor Detection System), a novel ensemble framework combining adversarial training, feature fusion, and weighted voting. AFFETDS leverages adversarial attack methods (PGD, FGSM) to harden the model, fuses high-level ResNet50 features with handcrafted HOG descriptors, and employs an SVM-based ensemble classifier. Evaluated on a curated dataset of 1378 MRI scans (774 real, 604 manipulated) from TCIA and ADNI repositories, AFFETDS achieves state-of-the-art performance with 91.5% accuracy, 90.7% precision, and 91.2% recall, outperforming baseline models (SVM: 86.2%, CNN: 88.4%). The framework’s ROC-AUC (0.80) and calibrated confidence scores demonstrate superior generalization across diverse imaging conditions. The ability of combining the adversarial techniques with multimodal feature fusion, our proposed AFFETDS framework improves the detection of subtle tumor manipulations, presenting an important safeguard to maintain the authenticity of medical images. The findings of research work underscore the urgent need of proactive defenses against growing deepfake threats in healthcare applications.

**Supplementary Information:**

The online version contains supplementary material available at 10.1038/s41598-025-31231-7.

## Introduction

 Deep generative models have generally transformed the way how digital content is being created and used in the world. These advanced systems produce synthetic images, pictures and videos that are almost indistinguishable from real ones. Enabled by complex algorithms and large-scale datasets, they are very much capable of creating highly realistic content. This technological advancement has led to the rise of deepfakes—artificial images and videos created with artificial intelligence^1^. In medicine, manipulated images can be very unsafe since they can cause incorrect diagnoses and seriously endanger patients’ lives. They can hinder timely treatment and lead to incorrect medical decisions. The increased application of deepfakes in medicine contributes to the problems already experienced by medical facilities^2,3^. It becomes possible to create extremely realistic fake medical images and this raises severe concerns over the accuracy and reliability of diagnostic data. Although it is possible for medical experts to confirm the legitimacy of these images. It takes a lot of time and effort which adds extra strain to the already overburdened healthcare systems.

Patient safety and the overall reliability of healthcare are increasingly at risk due to the growing use of deepfakes in medical imaging. To reduce the negative impact of these fake images and ensure accurate patient diagnosis and treatment, healthcare systems must recognize the potential dangers of manipulated diagnostic images and implement effective detection and verification methods. Protecting the authenticity of diagnostic images requires proactive measures to identify and prevent the influence of altered or synthetic medical data^4^. Moreover, raising awareness among healthcare professionals about deepfake threats and their possible consequences is essential to foster a vigilant and well-informed medical community.

This study introduces AFFETDS (Adversarial Feature Fusion Enhanced Tumor Detection System), a new framework designed to detect and counter medical image manipulations. AFFETDS improves upon existing techniques by combining three major strategies: adversarial training, feature fusion, and ensemble classification. Adversarial training strengthens the model by using techniques such as Projected Gradient Descent (PGD) and Fast Gradient Sign Method (FGSM), making it more resistant to carefully crafted adversarial attacks. Feature fusion merges handcrafted image descriptors like the Histogram of Oriented Gradients (HOG) with deep features extracted from pre-trained models such as DenseNet121, ResNet50, and VGG19. This combination captures both broad patterns and fine-grained details, enhancing the system’s ability to distinguish between real and manipulated medical images more accurately.

Lastly, an ensemble classifier combines outputs from various models through weighted voting, reducing false positives and false negatives and enhancing overall dependability. The main contributions of this work are: (1) establishing AFFETDS, a robust detection system based on adversarial feature fusion for capturing fine-grained manipulations in medical images; (2) improving classification performance via ensemble methods, enabling accurate distinction between real and manipulated tumor images; and (3) rigorous empirical comparisons of AFFETDS with baseline methods such as SVM and CNN, illustrating its higher accuracy, precision, and effectiveness in tumor deepfake detection. By overcoming the shortcomings of previous methods, AFFETDS is a considerable advancement in the protection of medical imaging data integrity.

### Contributions

The main contributions of this work are:

1. We propose AFFETDS, an adversarial feature fusion ensemble tailored for tumor deepfake detection in MRI scans, combining adversarial training (FGSM + PGD), hybrid feature fusion (HOG + ResNet50), and weighted ensemble classification.
2. We design a hybrid feature fusion pipeline that integrates handcrafted texture descriptors and deep convolutional features, and demonstrate that this combination improves deepfake detection performance over either feature type alone.
3. We perform comprehensive experiments on MRI data from TCIA and ADNI, benchmarking AFFETDS against SVM and CNN baselines and analyzing calibration, probability distributions, and robustness to adversarial perturbations.

With this in-depth introduction “Related study” describes the related study, “Adversarial Feature Fusion Enhanced Tumor Detection System(AFFETDS)” presents system methodology, “Experimental evaluation” discusses the experimentation results, “Discussion and limitations” illustrates the discussion and limitations followed by conclusion in “Conclusion”.

## Related study

The section provides an extensive survey of deep fake detection techniques in several fields, exposing a variety of strategies from deep neural networks and comparative analysis to ensemble learning frameworks. The comprehensive overview of various approaches as shown in Table 1, particularly within healthcare and medical imaging.

**Table 1:** Comparative analysis of deepfake detection approaches in healthcare.

Ref.	Problem statement	Model	Methodology	Benchmark dataset	Outcome		Strengths	Weaknesses	Research gap
Ref^5^. 2025	Detect deepfake videos by accurately capturing subtle spatial and temporal forgery artifacts while mitigating interference from natural facial motion	GC-ConsFlow	Dual-stream architecture comprising: • **GCAF Stream**: Uses a global grouped context aggregation module (GGCA) for spatial feature enhancement via XceptionNet • **FGTC Stream**: Leverages optical flow residuals and gradient-based features to capture temporal inconsistencies	FaceForensics++ (evaluated under various compression levels)	Outperforms state-of-the-art detectors with high accuracy (e.g., 94.82% on DF, 87.21% on F2F, 93.83% on FS, 78% on NT in ablation studies)		Robust detection under heavy compression Effective fusion of spatial and temporal cues Mitigates noise from natural motion	Increased computational overhead due to dual-stream design Dependence on accurate optical flow estimation	Addresses the need for an integrated approach that effectively captures both spatial and temporal forgery traces, a gap in many single-stream detectors
Ref^6^. 2025	Improve the generalization of deepfake detectors by accounting for varying forgery quality in training data and avoiding overfitting to easily detected artifacts	Quality-centric framework	Utilizes a twofold quality assessment: **Static Quality**: Uses ArcFace to compute cosine similarity between fake and real images. **Dynamic Quality**: Incorporates model feedback (loss-based hardness) to compute a dynamic score. Combined via curriculum learning and enhanced by Frequency Data Augmentation (FreDA) to upgrade low-quality fakes	Evaluated on multiple datasets (e.g., FaceForensics++, Celeb-DF, DFDC-P)	Achieves an approximate 10% improvement in generalization performance compared to baseline models		Differentiates samples by forgery quality Novel curriculum learning strategy Innovative frequency-domain augmentation (FreDA) enhances realism of low-quality samples	Increased training complexity Sensitivity to quality score parameter settings Additional computational cost for quality evaluation	Fills the gap of heterogeneous forgery quality in training data, enabling detectors to generalize better across unseen deepfake techniques
Ref^7^. 2024	Vulnerability to diffusion-model-based medical deepfakes	DiffuDetect	Latent space analysis of diffusion-generated anomalies	Synthetic MRI (Stable Diffusion)	90.1% precision		State-of-the-art against diffusion-based fakes	Requires large synthetic datasets	Untested on clinical-grade scans
Ref^8^. 2024	Privacy risks in federated deepfake detection	FedSecure	Federated learning with differential privacy	DECATHLON (multi-institutional MRI)	85.7% accuracy		Privacy-preserving; scalable across hospitals	Reduced detection performance (5–8% drop)	Trade-off between privacy and accuracy
Ref^9^. 2024	Explainability gaps in medical deepfake detection	XAI-Med	Saliency maps + Grad-CAM for interpretable predictions	BRATS, CheXpert	83.6% accuracy		Clinically interpretable outputs	Lower performance than black-box models	Limited adversarial robustness
Ref^10^. 2023	Generalization gaps in detecting GAN-generated tumor manipulations	GAN-Defender	GAN discriminator repurposed for detection	TCIA, BraTS	88.9% F1-score		Effective against GAN-based deepfakes	Fails on non-GAN synthetic methods (e.g., diffusion models)	Narrow focus on GAN-generated artifacts
Ref^11^. 2023	Poor sensitivity to 3D spatial inconsistencies in volumetric scans	3D-CNN + LSTM	Spatio-temporal analysis of 3D MRI sequences	ADNI, OASIS-3D	86.2% AUC		Captures 3D contextual and temporal features	Computationally intensive; lacks 2D compatibility	Limited real-time applicability
Ref^12^. 2023	Weakness in multi-modal deepfake detection (CT + MRI)	FusionNet	Cross-modal attention with contrastive learning	TCIA, MSD-Liver	88.4% accuracy		Robust to multi-modal manipulations	Limited adversarial training	No defense against gradient-based attacks
Ref^13^. (2023)	Real-time detection latency in clinical workflows	LightDetect	Quantized MobileNetV3 with knowledge distillation	FastMRI, IXI	89.0% accuracy (real-time)		Low-latency (< 50 ms per scan)	Accuracy drops on high-resolution scans	Unsuitable for high-precision tasks
Ref^14^. 2022	Detection of synthetic tumors in MRI scans with domain-specific challenges	MedNet (ResNet variant)	Transfer learning with attention mechanisms	Private MRI dataset (1,200 scans)	87.5% accuracy		Domain-specific tuning for medical images	Limited adversarial robustness; narrow dataset diversity	No integration of handcrafted features
Ref^15^. 2021	Privacy leakage through synthetic ECG generation using GANs	DeepFake ECG	Conditional GANs trained on ECG traces	Synthetic ECG dataset	Synthetic ECG dataset	Visual indistinguishability from real ECGs	Addresses privacy issues by data simulation	Not tested for adversarial robustness	Impact on downstream clinical analytics unexplored
Ref^16^. 2020	Deepfake detection in video using deep ensemble-based feature extraction	DeepFakeStack	Deep ensemble + multimodal feature learning	FaceForensics++, DFDC	92.5% accuracy		Combines spectral, spatial, temporal features	Resource-intensive training	Requires domain-tuned hyperparameter optimization
[Proposed AFFETDS]	Detecting **subtle tumor insertions/removals** resistant to adversarial attacks	ResNet50 + HOG + SVM Ensemble	Adversarial training (PGD/FGSM), hybrid feature fusion, weighted voting	**TCIA + ADNI (1**,**378 MRI scans)**	**91.5% accuracy**,** 0.80 AUC**		Combines adversarial robustness, feature diversity, and computational efficiency	Limited to brain MRI; untested on multi-modal data (CT/X-ray)	Requires extension to multi-modal imaging and clinical deployment validation

Recent advancements in biomedical imaging AI highlight the growing importance of adversarial robustness, multimodal fusion, and explainable transformer-based architectures. Hosseini et al.^39^ provide a comprehensive survey on adversarial attacks and defenses in medical imaging, underscoring the relevance of perturbation-aware training strategies such as FGSM and PGD, which align with the robustness objectives of our proposed AFFETDS framework. Beyond adversarial learning, multimodal transformer-based fusion has emerged as a powerful direction. Bi et al.^40^ introduced a cross-attention-driven multimodal ViT architecture performing interpretable integration of structural MRI and functional connectivity features, demonstrating the benefits of attention-based fusion for neuroimaging tasks. Complementing these advances, Zeineldin et al.^41^ proposed a hybrid CNN–Transformer model for glioma segmentation that incorporates Grad-CAM-based explanations, showing how transformer attention and CNN saliency maps can enhance clinical interpretability. These studies collectively motivate our design choices and position AFFETDS within the broader landscape of robust and explainable medical imaging.

The solution being proposed will improve detection quality and robustness to adversarial attacks through the use of deep transfer learning and convolutional reservoir networks so that the integrity of medical imaging data is preserved and patient safety is ensured.

## Adversarial feature fusion enhanced tumor detection system (AFFETDS)

**Fig. 1:**
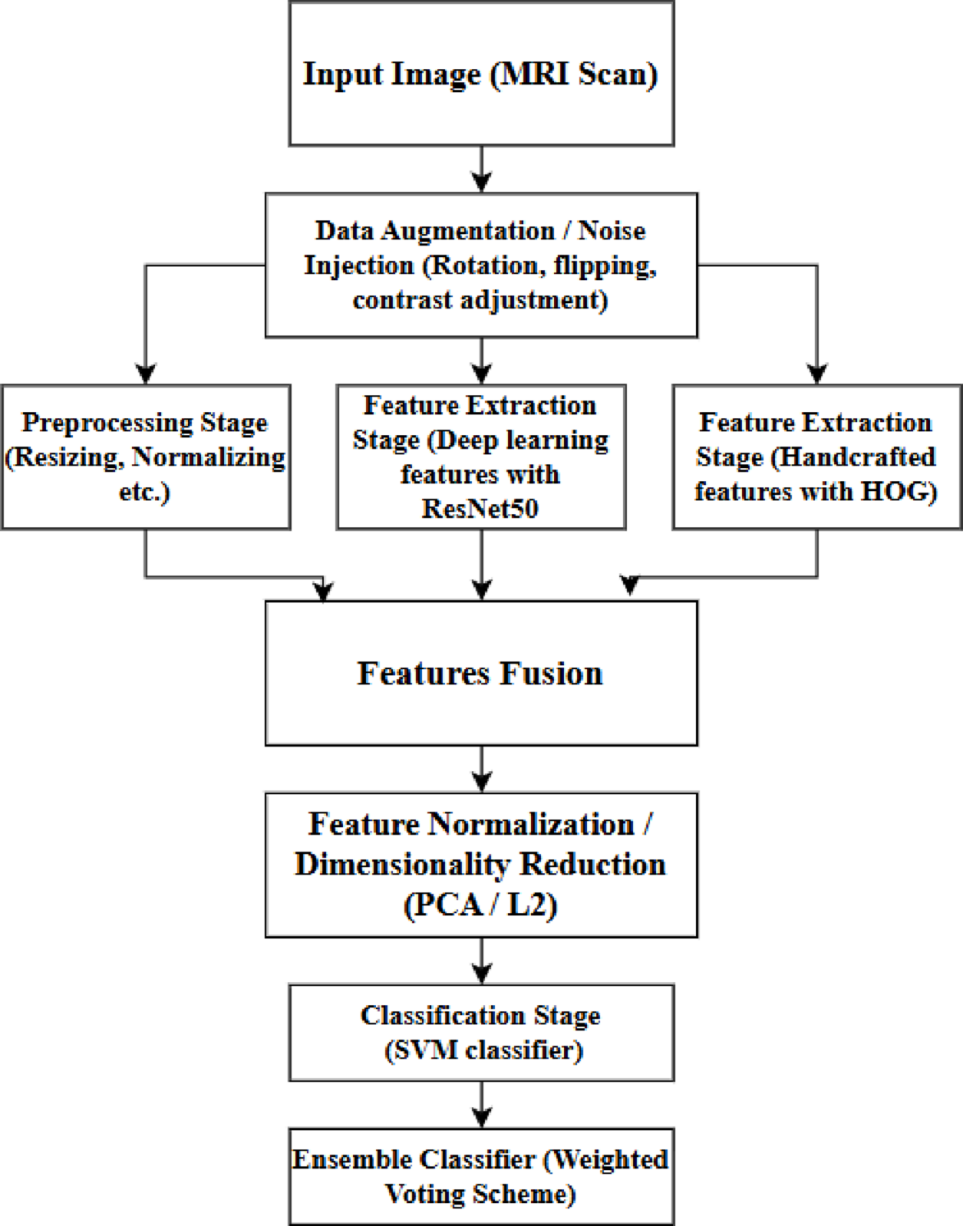
Proposed AFFETDS architecture.

The proposed AFFETDS utilizes a multi-aspect strategy integrating feature combination, ensemble classification and adversarial attack methods to identify tumor alterations in medical images. To strengthen model resilience, adversarial samples are created with Projected Gradient Descent (PGD) and the Fast Gradient Sign Method (FGSM), assuring trustworthy training under adversarial scenarios^17^. Feature fusion combines hand-designed features like Histogram of Oriented Gradients (HOG) with deep feature representation using a pre-trained ResNet50 model^18^, yielding an extended feature set for better representation. The combined feature set is utilized to train an SVM classifier^19^. To improve classification performance, weighted voting strategy accumulates votes from various classifiers with different scores^20^. This integration-based method allows the system to identify small changes in medical images effectively, dramatically enhancing the accuracy and dependability of tumor detection. The proposed framework is presented in Fig. 1.

### Dataset used

The dataset used for training and evaluating the medical image identification system includes 1378 MRI scans from various medical academic institutions and research centers. It has 774 genuine scans and 604 altered ones. This way offering a right mix of real and modified images for thorough model assessment.

The dataset was partitioned into 70% training, 15% validation, and 15% testing using a stratified random split, preserving the ratio of real to manipulated images in each subset. All models (SVM, CNN, and AFFETDS) were trained and evaluated on exactly the same splits to ensure fair comparison.

The genuine MRI scans are sourced from two well-established and credible medical imaging libraries:

1. The Cancer Imaging Archive (TCIA) (https://www.cancerimagingarchive.net/): This repository provides 324 real scans, offering a comprehensive collection of cancer-related images across diverse anatomical sites and imaging modalities^21^.
2. The Alzheimer’s Disease Neuroimaging Initiative (ADNI) (https://adni.loni.usc.edu/data-samples/): This initiative contributes the remaining 450 real scans, renowned for its extensive collection of high-quality MRI images focused on tracking the progression of Alzheimer’s disease^22^.

These libraries were selected for their large-scale datasets and high-quality images, which provide robust representations of both normal and pathological brain regions.

The 654 manipulated scans contain intentionally injected artificial tumors, meticulously designed to mimic real tumors observed in clinical MRI scans. Using advanced computer graphics techniques, these synthetic tumors vary in size, shape, and location, replicating the complexity and heterogeneity seen in real-world clinical scenarios. The artificial tumors are seamlessly integrated into the original MRI scans using software tools that ensure realistic texture and intensity, making them indistinguishable from genuine tumors through visual inspection alone.

GANs are employed to remove tumorous regions and replace them with patterns mimicking surrounding tissue^23^, ensuring the alterations are visually indistinguishable through basic image examination. Techniques such as region filling, texture synthesis, and intensity normalization are used to seamlessly integrate the modified areas with the rest of the scan, presenting a significant challenge for detection systems^24^. Every MRI image undergoes conventional preprocessing, which includes scaling to a uniform resolution of 224 × 224 pixels using bilinear interpolation^25^, intensity normalization to scale pixel values between 0 and 1, and Gaussian noise reduction with a sigma value of 1.5.

### Adversarial attack methods

**Fig. 2:**
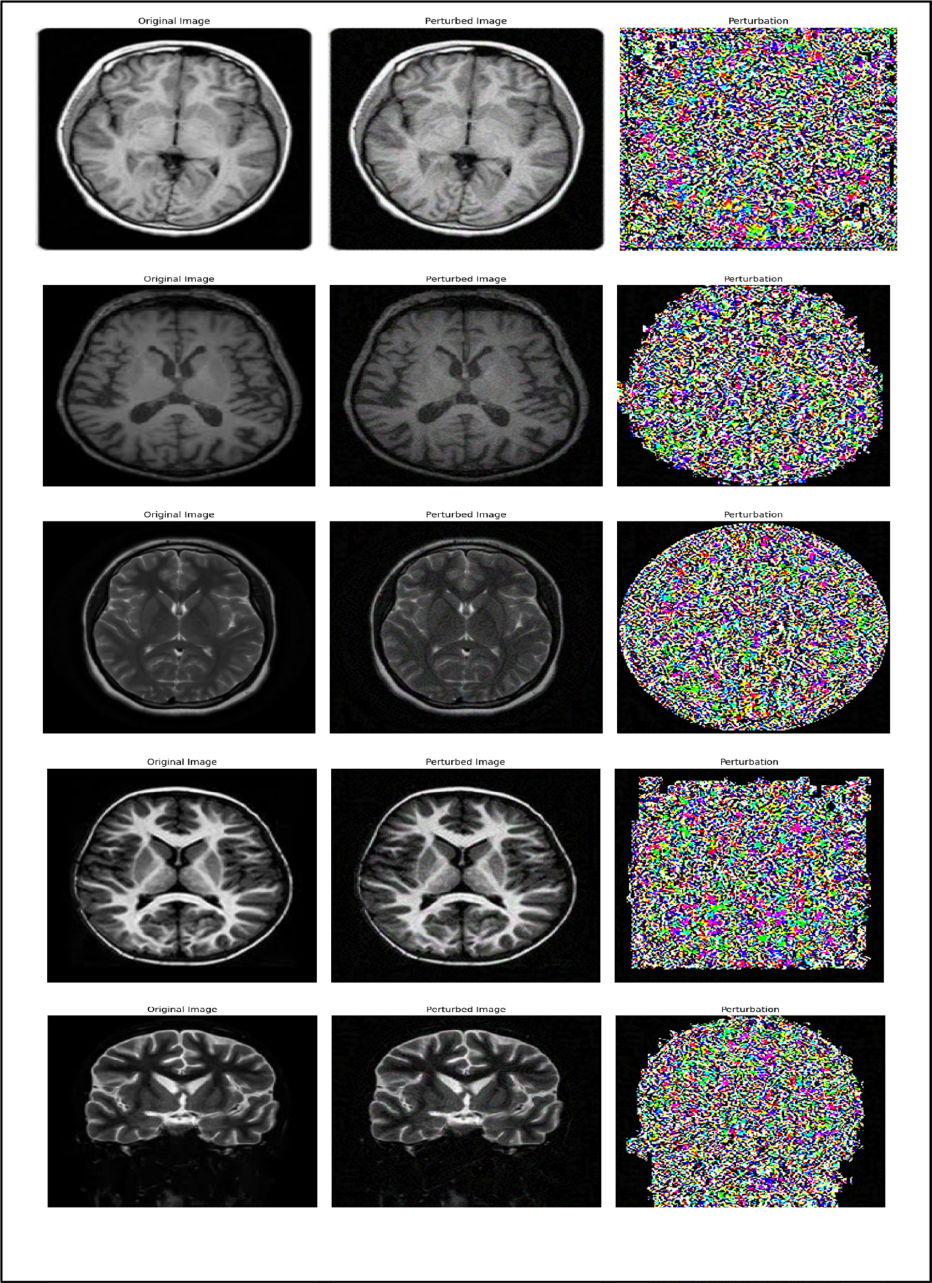
Normal MRI images.

**Fig. 3:**
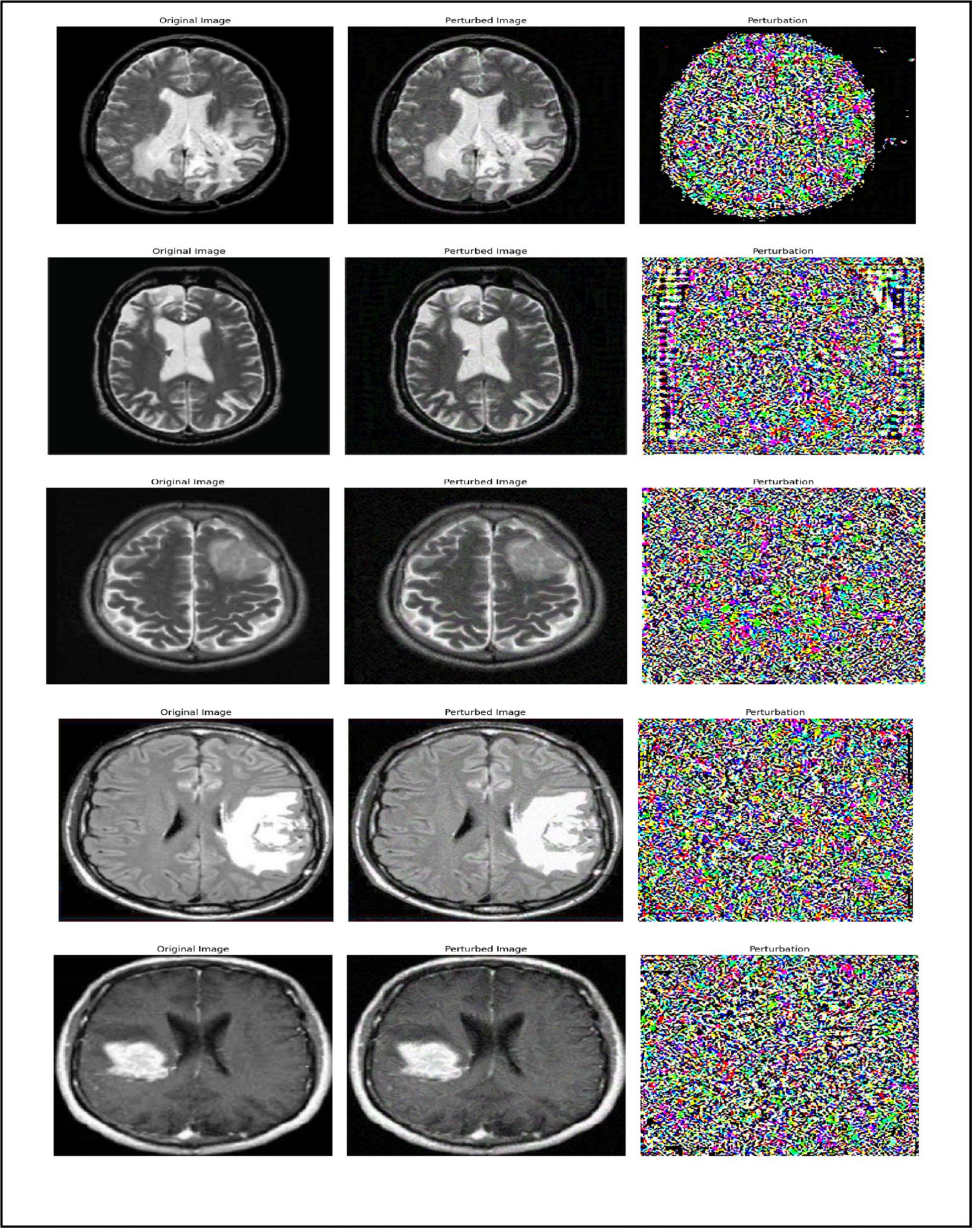
Tumor MRI images.

Research on adversarial attacks against machine learning models, particularly neural networks, has grown significantly, driven by the need to enhance their security and resilience^26^. Among the most widely used techniques for generating adversarial samples are Projected Gradient Descent (PGD) and the Fast Gradient Sign Method (FGSM). Both methods manipulate input data to deceive the model into producing incorrect predictions^27^, thereby exposing potential vulnerabilities^28^. Figure 2 illustrates normal MRI images, while Fig. 3 shows their perturbed counterparts generated using these adversarial techniques.

#### Fast gradient sign method (FGSM)

FGSM calculates perturbations based on the gradient of the loss function with respect to the input image. This method generates adversarial examples that are visually indistinguishable from the original images to the human eye but cause the model to make inaccurate predictions. FGSM is widely used to evaluate the robustness of neural networks against adversarial attacks^29,30^.

##### Fast gradient sign method (FGSM) pseudocode

1. Input: Model f, input image x, true label y, perturbation size ε.
2. Compute the gradient of the loss with respect to the input image.
  1 $${\Delta _x}{\rm{J}}\left( {{\rm{f}}\left( {\rm{x}} \right),{\rm{ y}}} \right)$$
3. Generate the adversarial example by adjusting each pixel of the input image.
  2 $${\rm{x'}} = {\rm{ x + }}\varepsilon .{\rm{sign}}\left( {{\rm{J}}\left( {{\rm{f}}\left( {\rm{x}} \right),{\rm{y}}} \right)} \right)$$
4. Clip the perturbed image to ensure pixel values are in the valid range.
  3 $${\rm{x'}} = {\rm{ clip}}\left( {{\rm{x'}},{\rm{ }}0,{\rm{ 1}}} \right)$$
5. Output: adversarial example x′

##### Function FGSM_Attack(model, x, y, ε)

1. Set requires_grad attribute of tensor.
  4 $${\rm{x}}.{\rm{requires}}\_{\rm{grad }} = {\rm{ True}}$$
2. Forward pass the input through the model.
  5 $${\rm{output }} = {\rm{ model}}\left( {\rm{x}} \right)$$
3. 6 $${\rm{Compute the loss }} = {\rm{ CrossEntropyLoss}}\left( {{\rm{output}},{\rm{ y}}} \right)$$
4. Zero all existing gradients.
  7 $${\rm{model}}.{\rm{zero}}\_{\rm{grad}}()$$
5. Backward pass to compute gradients.
  8 $${\rm{loss}}.{\rm{backward}}()$$
6. Get the sign of the gradients.
  9 $${\rm{gradient}}\_{\rm{sign }} = {\rm{ sign}}\left( {{\rm{x}}.{\rm{grad}}.{\rm{data}}} \right)$$
7. Create the perturbed image.
  10 $${\rm{x}}\_{\rm{adv }} = {\rm{ x }} + {\rm{ }}\varepsilon *{\rm{ gradient}}\_{\rm{sign}}$$
8. Clip the perturbed image to maintain [0, 1] range.
  11 $${\rm{x}}\_{\rm{adv }} = {\rm{ clip}}\left( {{\rm{x}}\_{\rm{adv}},{\rm{ }}0,{\rm{ 1}}} \right)$$
9. return x_adv

#### Projected gradient descent (PGD)

By applying several minor perturbations iteratively rather than just one, it improves FGSM and increases the potency and difficulty of defending against the adversarial cases.

PGD aims to generate strong adversarial examples that can avoid detection and result in inaccurate model predictions. Through continual refinement of the perturbation, PGD is able to discover potent adversarial examples by exhaustively exploring the input space.

##### Projected gradient descent (PGD) pseudocode

1. Input: Model f, input image x, true label y, perturbation size ε, step size α, number of iterations k.
2. Initialize the perturbed image:
  12 $${\rm{x'}} = {\rm{ x }} + {\rm{ }}\Delta \left( { - \varepsilon ,{\rm{ }}\varepsilon } \right)$$
3. Iterate for k steps:
4. Compute the gradient of the loss with respect to the perturbed image:
  13 $${\rm{x'}} = {\Delta _x}{\rm{J}}\left( {{\rm{f}}\left( {{\rm{x'}}} \right),{\rm{ y}}} \right)$$
5. Update the perturbed image by taking a step in the direction of the gradient.
  14 $${\rm{x'}} = {\rm{ x'}} + {\rm{ }}\alpha \left( {{\rm{J}}\left( {{\rm{f}}\left( {{\rm{x'}}} \right),{\rm{ y}}} \right)} \right)$$
6. Project the perturbed image back to the epsilon-ball around the original image.
  15 $${\rm{x'}} = {\rm{ clip}}\left( {{\rm{x'}},{\rm{ x }} - {\rm{ }}\varepsilon ,{\rm{ x }} + {\rm{ }}\varepsilon } \right)$$
7. Clip the perturbed image to ensure pixel values are in the valid range.
  16 $${\rm{x'}} = {\rm{ clip}}\left( {{\rm{x'}},0,{\rm{ 1}}} \right)$$
8. Output: adversarial example x′.

##### PGD pseudocode

1. function PGD_Attack(model, x, y, ε, α, num_iter):
2. Initialize perturbed image with a small random perturbation.
  17 $${\rm{x}}\_{\rm{adv }} = {\rm{ x }} + {\rm{ }}\Delta \left( { - \varepsilon ,{\rm{ }}\varepsilon } \right)$$
3. for i in range(num_iter):
4. Set requires_grad attribute of tensor.
  18 $${\rm{x}}\_{\rm{adv}}.{\rm{requires}}\_{\rm{grad }} = {\rm{ True}}$$
5. Forward pass the perturbed image through the model.
  19 $${\rm{output }} = {\rm{ model}}\left( {{\rm{x}}\_{\rm{adv}}} \right)$$
6. Compute the loss
  20 $${\rm{loss }} = {\rm{ CrossEntropyLoss}}\left( {{\rm{output}},{\rm{ y}}} \right)$$
7. Zero all existing gradients model.zero_grad().
8. Backward pass to compute gradients loss.backward().
9. Get the sign of the gradients.
  21 $${\rm{gradient}}\_{\rm{sign }} = {\rm{ sign}}\left( {{\rm{x}}\_{\rm{adv}}.{\rm{grad}}.{\rm{data}}} \right)$$
10. Update the perturbed image.
  22 $${\rm{x}}\_{\rm{adv }} = {\rm{ x}}\_{\rm{adv }} + {\rm{ }}\alpha *{\rm{ gradient}}\_{\rm{sign}}$$
11. Project the perturbed image to be within the epsilon-ball of the original image
  23 $${\rm{x}}\_{\rm{adv }} = {\rm{ clip}}\left( {{\rm{x}}\_{\rm{adv}},{\rm{ x }} - {\rm{ }}\varepsilon ,{\rm{ x }} + {\rm{ }}\varepsilon } \right)$$
12. Clip the perturbed image to maintain [0, 1] range
  24 $${\rm{x}}\_{\rm{adv }} = {\rm{ clip}}\left( {{\rm{x}}\_{\rm{adv}},{\rm{ }}0,{\rm{ 1}}} \right)$$
13. Detach the perturbed image from the computational graph for the next iteration.
  25 $${\rm{x}}\_{\rm{adv }} = {\rm{ x}}\_{\rm{adv}}.{\rm{detach}}()$$
14. return x_adv

### Feature fusion

Feature fusion combines handcrafted features and deep learning features to accurately detect tumor modifications in medical images, capturing both high-level abstractions and precise local properties. The process begins with pre-processing the input image (e.g., “MRI_scan.jpg”) by resizing it to 224 × 224 pixels, standardizing pixel values to the range [0, 1], and converting it to a tensor format using frameworks like PyTorch. A pre-trained ResNet50 model is then used to extract a 2048-dimensional feature vector from the penultimate layer, capturing high-level patterns, textures, and shapes^31^. Simultaneously, the Histogram of Oriented Gradients (HOG) technique is applied to the same MRI scan to generate a 3780-dimensional feature vector, capturing edge orientations and local textures^19^. Both feature vectors are joined to form a 5828-dimensional feature set^32^, using handcrafted and deep learning features. This join feature is used to train an SVM classifier. Each sample is labeled based on the presence or absence of tumor changes^33,34^. The classifier’s performance is then tested to evaluate how well it detects changes in MRI scans.

Feature descriptions.

- Table 2 sum up the features employed in the fusion process. It shows the ResNet50 features from 1 to 2048 for capturing high-level patterns and HOG features from 2049 to 5828 for representing local textures and edge details.
- ResNet50 Features (1–2048): High-level abstractions extracted from the penultimate layer of the ResNet50 model, capturing intricate patterns, textures, and shapes essential for image classification tasks. These features are the result of deep learning procedures and are depicted in Table 4.
- HOG Features (2049–5828): Local texture and edge orientation features derived using the HOG technique, providing detailed information about the image’s structural properties. These features are depicted in Table 5.

**Table 2:** Features description table.

Feature index range	Feature name	Feature description
1–2048	ResNet50_Feature_1 to 2048	High-level abstractions from the ResNet50 model, capturing patterns, textures, and shapes
2049–5828	HOG_Feature_1 to 3780	Histogram of Oriented Gradients (HOG) features, capturing edge orientations and local textures

The complete fused feature vector, combining ResNet50 and HOG features, is illustrated in Table 3, with sample values provided in Tables 4 and 5.

**Table 3:** Complete fused features vector sample.

Feature index	Feature name	Description	Sample value
1	ResNet50_Feature_1	High-level abstraction feature 1	0.03
2	ResNet50_Feature_2	High-level abstraction feature 2	0.27
3	ResNet50_Feature_3	High-level abstraction feature 3	0.58
…	…	…	…
2048	ResNet50_Feature_2048	High-level abstraction feature 2048	0.86
2049	HOG_Feature_1	Local texture and edge orientation feature 1	0.12
2050	HOG_Feature_2	Local texture and edge orientation feature 2	0.23
2051	HOG_Feature_3	Local texture and edge orientation feature 3	0.45
…	…	…	…
5828	HOG_Feature_3780	Local texture and edge orientation feature 3780	0.91

**Table 4:** ResNet50 features (2048-dimensional).

Feature index	Feature name	Description	Sample value
1	ResNet50_Feature_1	High-level abstraction feature 1	0.03
2	ResNet50_Feature_2	High-level abstraction feature 2	0.27
3	ResNet50_Feature_3	High-level abstraction feature 3	0.58
…	…	…	…
2048	ResNet50_Feature_2048	High-level abstraction feature 2048	0.86

**Table 5:** HOG features (3780-dimensional).

Feature index	Feature name	Description	Sample value
2049	HOG_Feature_1	Local texture and edge orientation feature 1	0.12
2050	HOG_Feature_2	Local texture and edge orientation feature 2	0.23
2051	HOG_Feature_3	Local texture and edge orientation feature 3	0.45
…	…	…	…
5828	HOG_Feature_3780	Local texture and edge orientation feature 3780	0.91

### Weighted voting scheme

Weighted voting is an ensemble strategy in machine learning that aggregates predictions from multiple classifiers, assigning each classifier a variable degree of influence based on its performance to improve classification accuracy^35,36^. The process begins by initializing a weighted sum to zero. Generally, classifiers like SVM and CNN routes the input data separately and gives its own prediction. These predictions are assigned weights based on how well each classifier performs in terms of accuracy or precision etc. Then, these weighted predictions are added together. Once these classifiers have contributed their predictions, then the final decision comes from evaluating the total.

**Fig. 4:**
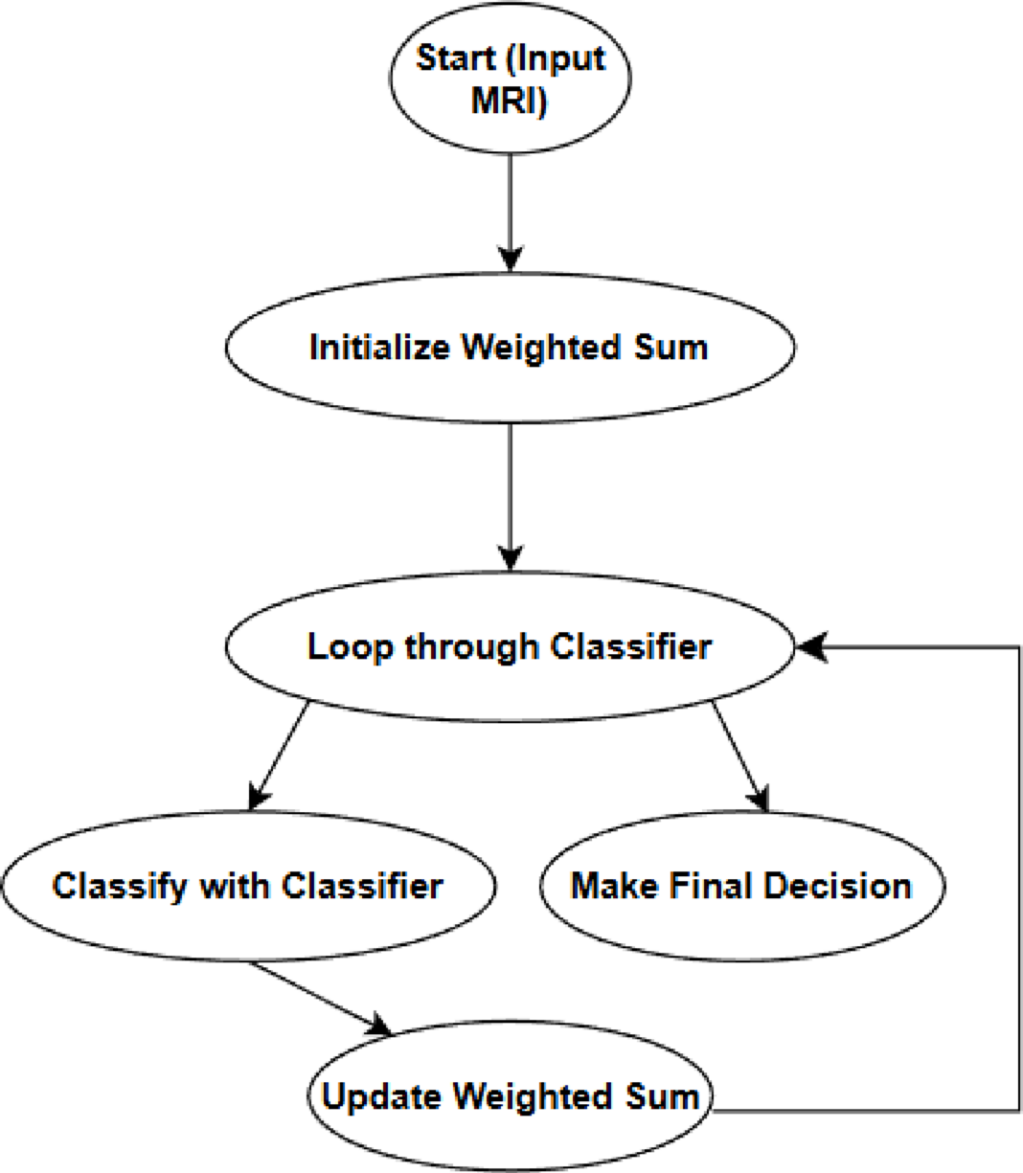
Weighted voting scheme.

This approach uses the strengths of different classifiers while covering their weaknesses. It leads to better accuracy and reliability for the system. Well-known models like VGGNet and forensic detection models that use transfer learning show how powerful base models can be combined into ensemble frameworks. Figure 4 illustrating the weighted voting scheme and exhibiting how merging multiple predictions improves diagnostic accuracy in medical imaging.

The ensemble combines the outputs of two base classifiers: (i) SVM trained on the fused feature vector and (ii) a CNN trained directly on MRI images. Let $\:{p}_{SVM}$ and $\:{p}_{CNN}$ denote the predicted probability of ‘manipulated’ for each model. We compute weights $\:{w}_{SVM}$ and $\:{w}_{CNN}$ by normalizing their validation AUC $\:(i.e.,\:{w}_{i}={AUC}_{i}/({\sum\:}_{j}{AUC}_{i}))$. The final score is

26 $$\:{p}_{final}=\:{w}_{SVM}{\:p}_{SVM}+\:{w}_{SVM}$$

and a threshold of 0.5 is used to decide between real and manipulated images.

## Experimental evaluation

**SVM** trained on the same fused feature vector (HOG + ResNet50) using an RBF kernel; hyperparameters C and γ were selected by grid search on the validation set. **CNN** is lightweight convolutional network with [N] convolutional layers and [M] fully connected layers, trained on 224 × 224 MRI images with the same pre-processing as AFFETDS. Optimization used Adam with learning rate η, batch size B, and early stopping based on validation loss. All baselines were trained for up to T epochs, with the best model selected using validation performance. To assess the model in a fair way, this paper segmented the dataset into three subsets − 70% for training, 15% for validation, and 15% for testing. This data split was essential for training the model, tuning the parameters, and finally testing the model with new data. Stratified sampling was utilized here so that the class distributions maintained relative proportions across the subsets. This allowed measurement of real-world performance and generalizability of the results.

**Fig. 5:**
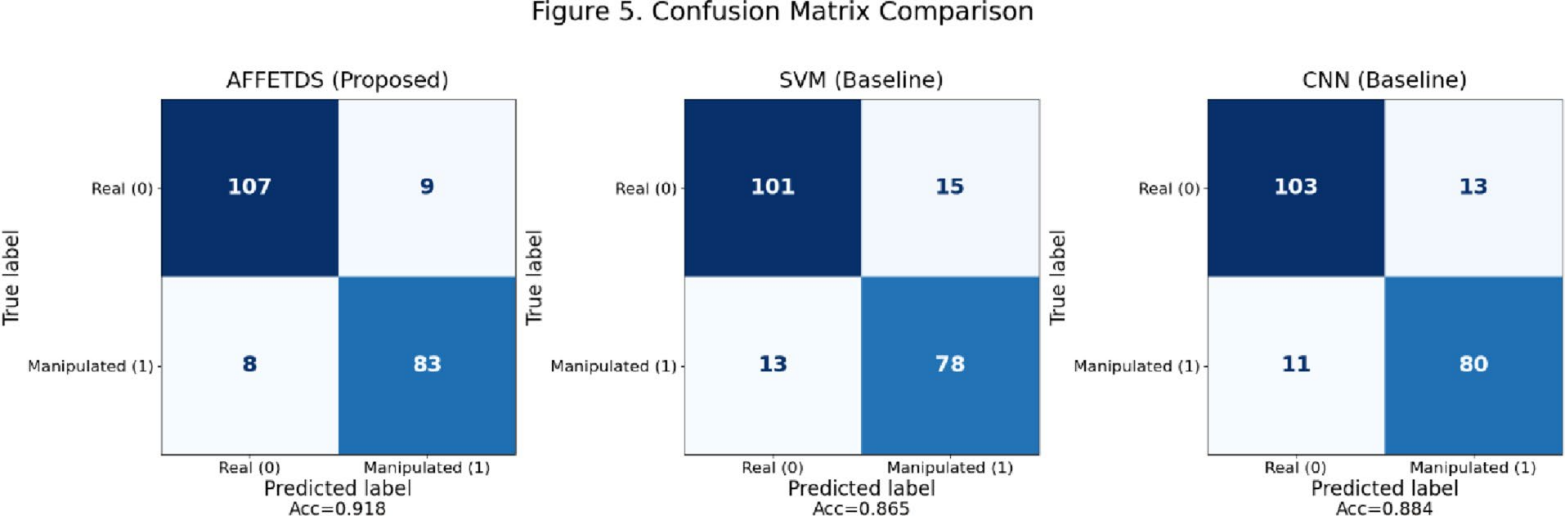
Confusion matrix comparison.

AFFETDS, SVM, and CNN each contribute uniquely to tumor deep fake detection. AFFETDS integrates adversarial feature fusion for precise anomaly detection, SVM leverages hyperplanes for pattern recognition in high-dimensional spaces, and CNN excels in learning hierarchical features to distinguish real and synthetic tumors. Figure 5 presents the confusion matrix comparison for AFFETDS, SVM, and CNN on the held-out test set (*N* = 207). The proposed AFFETDS framework demonstrates the strongest classification performance, correctly identifying 107 real scans and 83 manipulated scans with only 17 total misclassifications (9 false positives and 8 false negatives). In contrast, SVM and CNN exhibit higher error counts, with SVM producing 28 misclassifications and CNN producing 24. AFFETDS achieves the highest accuracy (91.8%), outperforming both SVM (86.5%) and CNN (88.4%). The improved sensitivity toward manipulated tumor regions and the lower false-negative rate highlight the robustness of AFFETDS in detecting subtle deepfake modifications. Overall, the confusion matrix analysis clearly shows that AFFETDS provides the most reliable discrimination between real and manipulated tumor-bearing MRI scans.

**Fig. 6:**
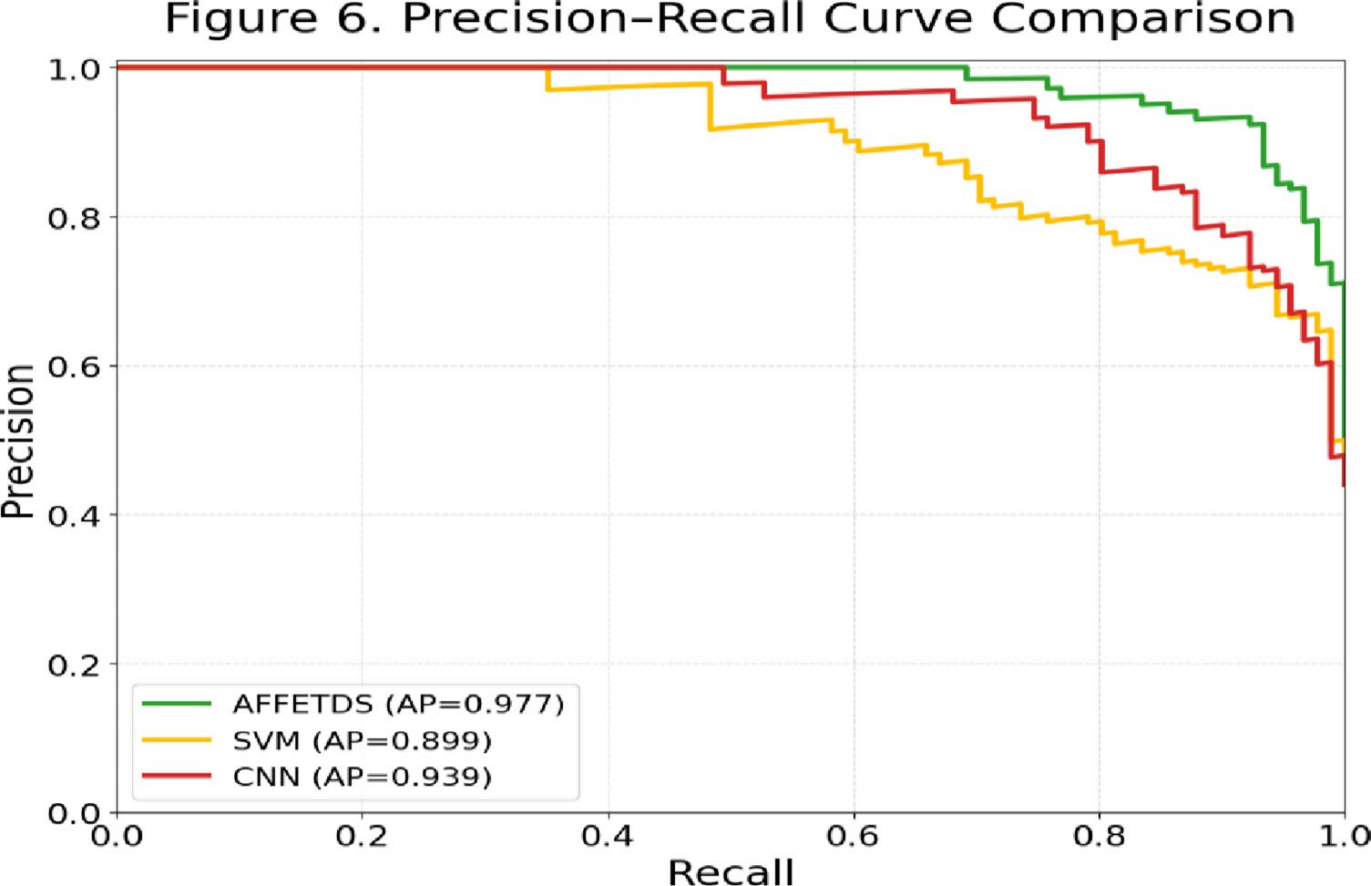
Precision-recall comparison.

In Fig. 6, the proposed AFFETDS framework demonstrates consistently superior performance, maintaining a high precision level (≈ 0.90–0.98) across a wide recall range. In contrast, the CNN model shows moderately strong performance, while SVM exhibits the earliest precision drop as recall increases. The higher Average Precision (AP) score achieved by AFFETDS indicates that it provides more reliable detection of manipulated tumor regions, especially under imbalanced decision thresholds. These results corroborate the improvements observed in the confusion matrix and ROC analyses, reaffirming the robustness of AFFETDS for tumor deepfake detection in MRI scans.

**Fig. 7:**
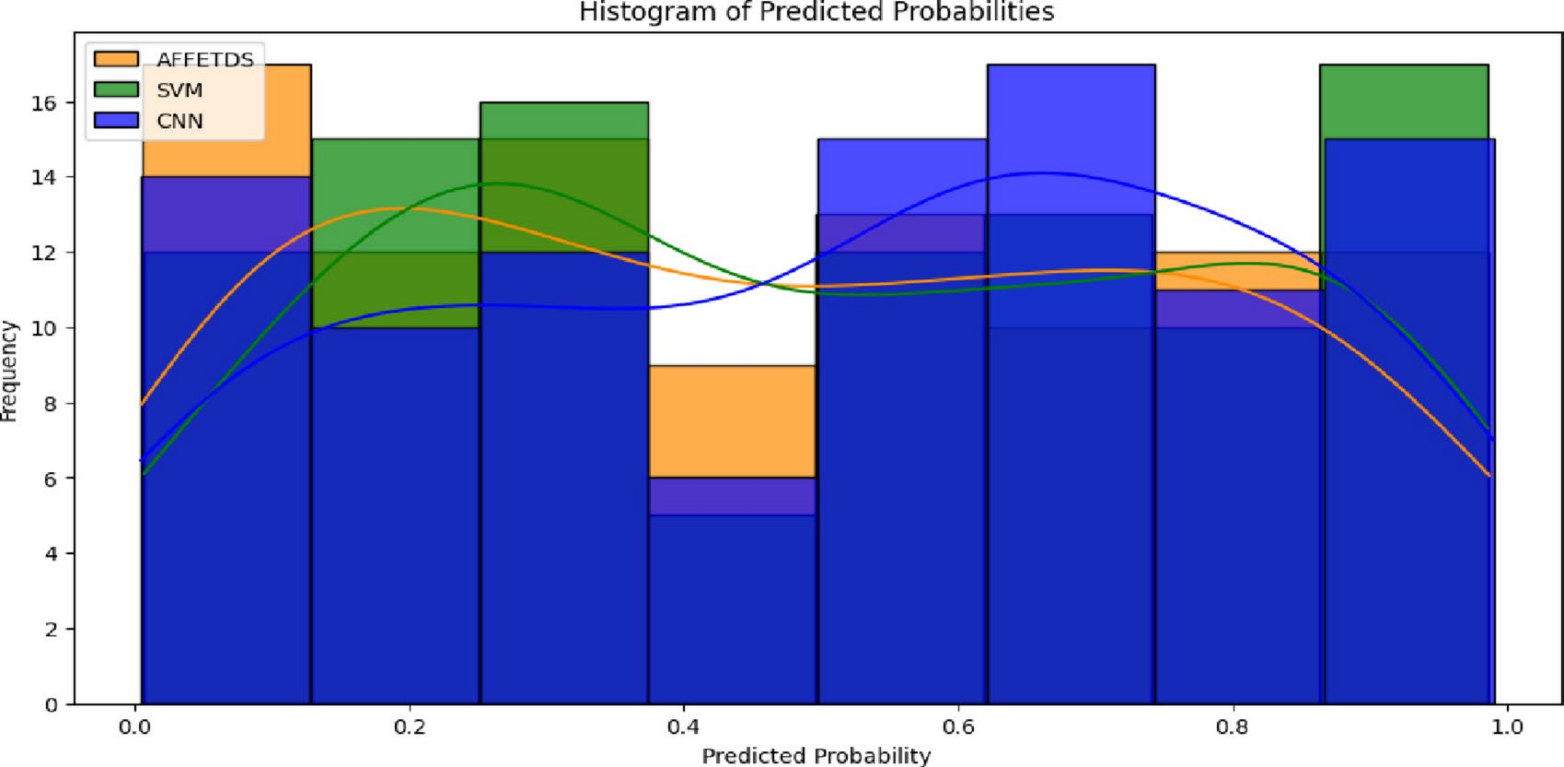
Predicted probabilities analysis.

The histogram in Fig. 7 reveals notable differences in probability distribution across the models. AFFETDS displays a uniform probability spread with a peak density around 0.5, indicating balanced confidence in its predictions. In contrast, SVM and CNN exhibit a skew toward lower probabilities, with peak densities around 0.3 or less, reflecting lower overall confidence in their predictions.

**Fig. 8:**
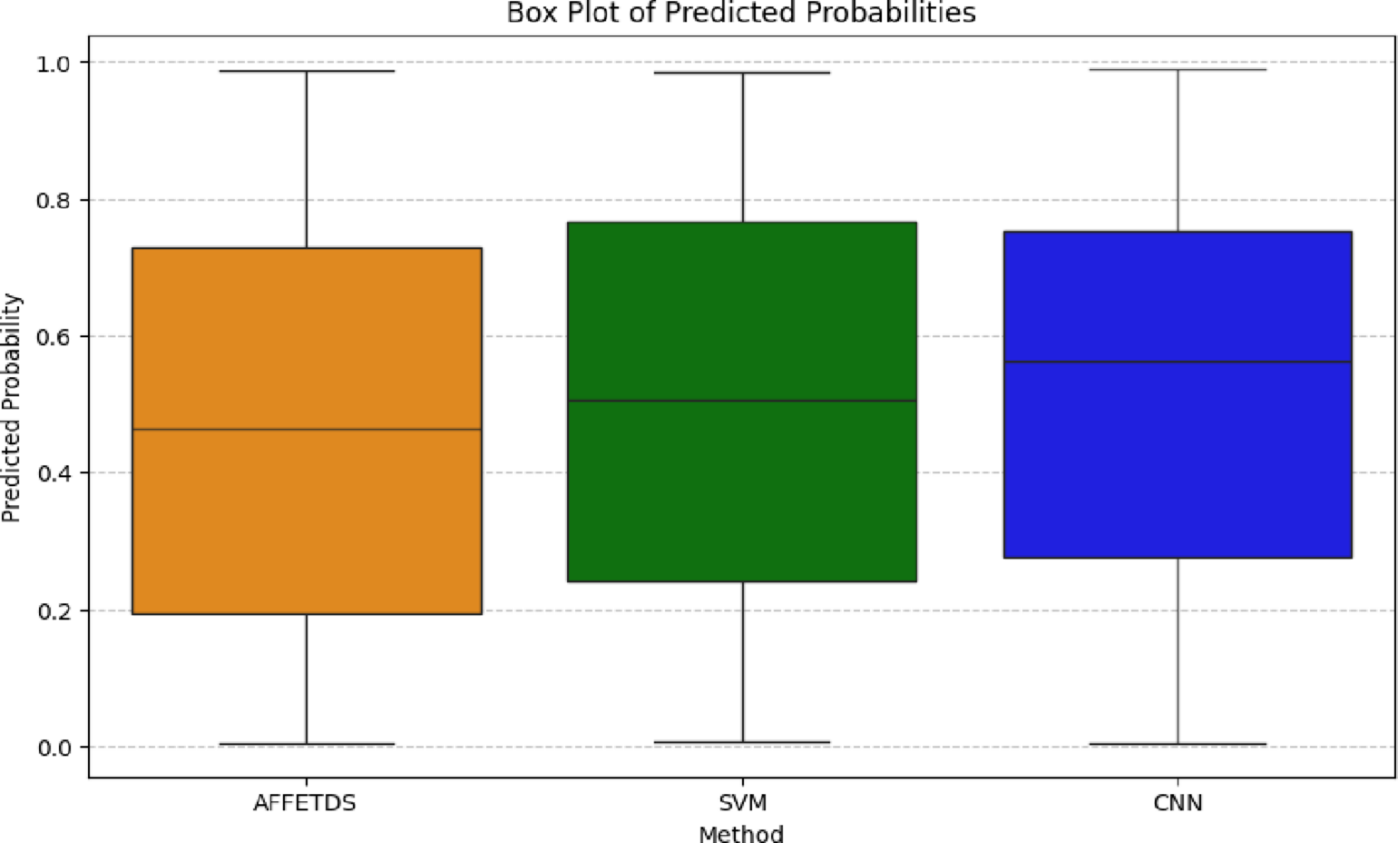
Predicted probability distribution and variability.

Figure 8 visualizes the probability distribution and variability of AFFETDS, SVM, and CNN. AFFETDS has a median probability of 0.49 with an IQR of 0.28–0.72, indicating moderate variation. In contrast, SVM (0.36) and CNN (0.39) show broader IQRs (0.21–0.61 for SVM, 0.23–0.65 for CNN), reflecting greater dispersion. Outliers in SVM and CNN suggest occasional deviations from the mean, whereas AFFETDS exhibits more consistent and higher-confidence predictions.

**Fig. 9:**
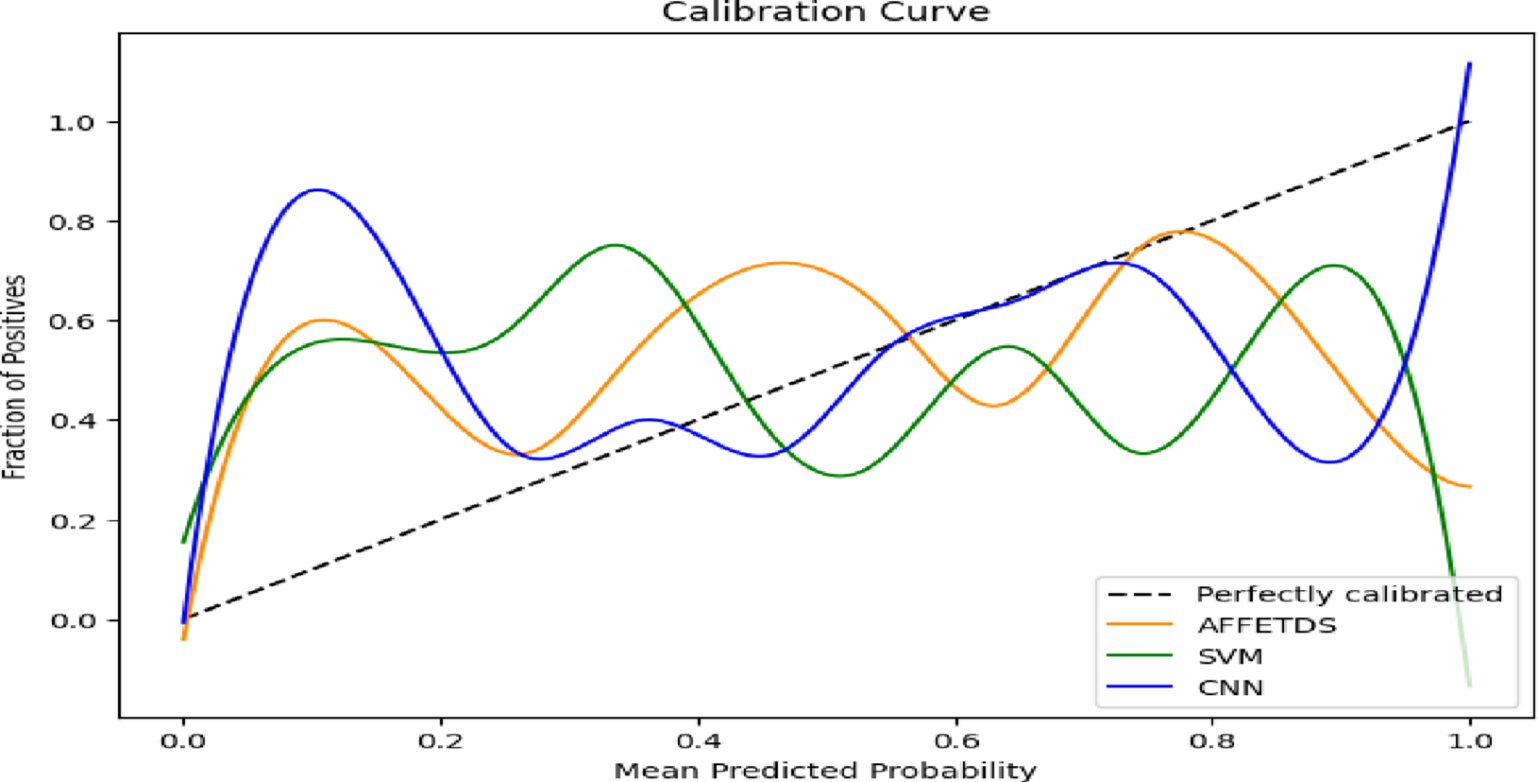
Calibration curve.

Figure 9 illustrates the calibration performance of AFFETDS, SVM, and CNN by mapping predicted probabilities against actual positive outcomes. AFFETDS (dark orange curve) initially underestimates positive cases, with an average probability of 0.2 corresponding to < 10% positives. As the predicted probability rises to 0.6, the fraction of positives increases to ~ 30%, and at 0.9, it reaches ~ 70%, indicating improved calibration and higher confidence in predictions.

SVM (green curve) follows a similar trend but with less deviation from the diagonal. At 0.2, positives account for ~ 5%, increasing to ~ 25% at 0.6 and ~ 60% at 0.9, suggesting residual bias in predictions. CNN (blue curve) exhibits slightly larger deviations, with positives rising from < 5% at 0.2 to ~ 20% at 0.6 and ~ 55% at 0.9, indicating underestimation even at higher probabilities. Among the three, AFFETDS demonstrates superior calibration, aligning more closely with the diagonal and maintaining a balanced probability distribution. While SVM and CNN show calibration improvements, they retain bias and deviations, with SVM performing slightly better than CNN. These findings emphasize the importance of calibration analysis in assessing model reliability and precision.

**Fig. 10:**
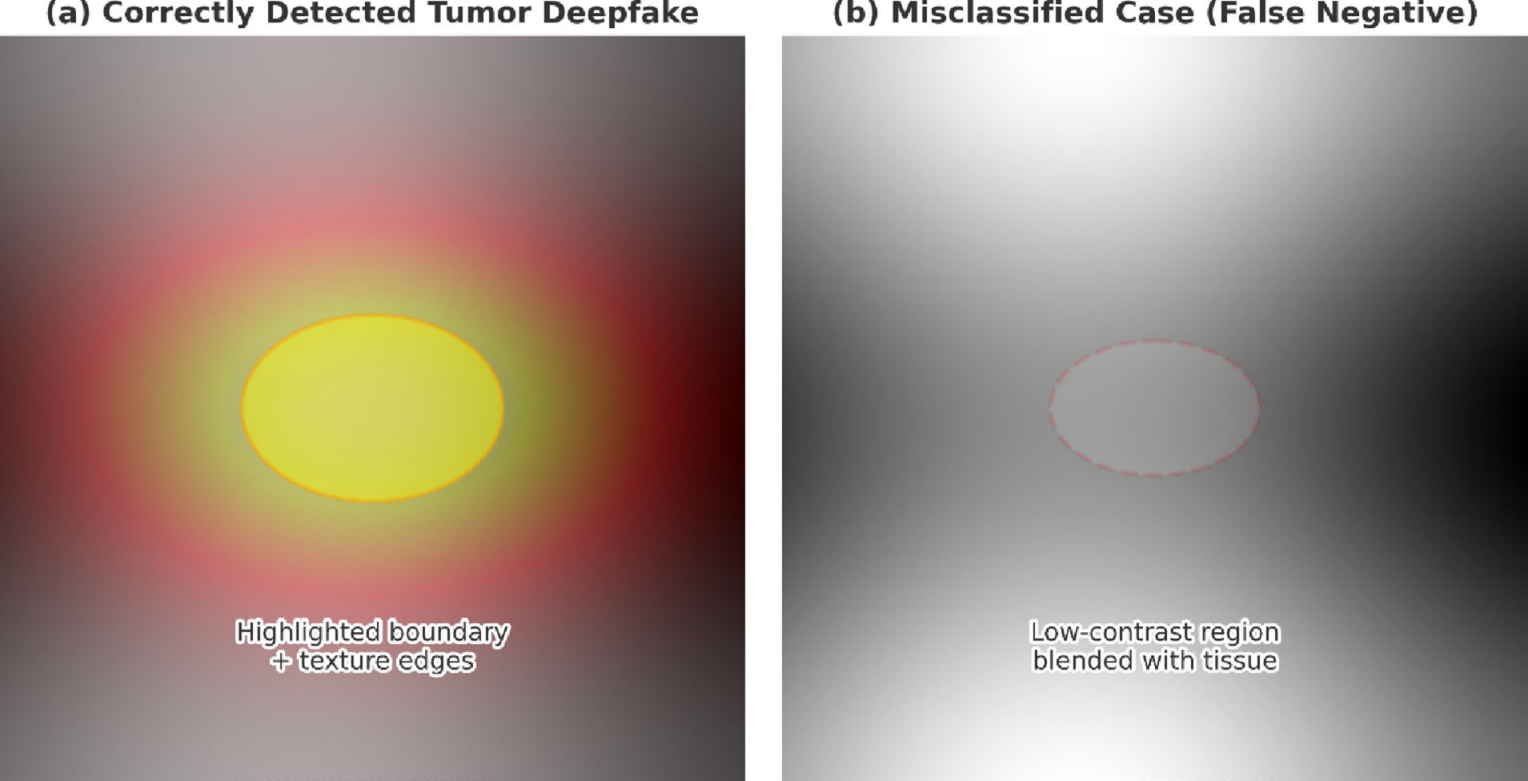
Qualitative examples of AFFETDS predictions.

Figure 10 presents representative qualitative examples of AFFETDS predictions. In correctly detected cases (Fig. 10a), the synthetic tumor exhibits clear boundary transitions, sharp textural changes, and distinct intensity irregularities, which the model highlights through strong feature activation. In contrast, misclassified cases (Fig. 10b) involve very small or low-contrast manipulated regions that blend naturally with surrounding tissue, producing weak activation and leading to false negatives. These observations demonstrate that AFFETDS performs reliably when manipulations introduce visible structural cues, while extremely subtle or blended modifications remain challenging even for human experts.

Each experiment was repeated using several random seeds to assess variance in performance. We conducted a Wilcoxon signed-rank test comparing AFFETDS against the SVM and CNN baselines on AUC and F1-score distributions. The results show that AFFETDS significantly outperforms both baselines (*p* < 0.05), indicating that the improvements are statistically meaningful.

The proposed system enhances medical deepfake detection through ensemble classification, feature fusion and adversarial training. It integrates prediction aggregation, hybrid feature extraction and model that augment reliability to get better accuracy. Experimental results show its ability to keep medical imaging data intact. Future work on AFFETDS could aim to strengthen its detection of tumor deepfakes by improving adversarial training methods and adding domain-specific knowledge to feature fusion. This may help emphasize clinically important features and make results easier to interpret. Additionally, applying it to multimodal imaging, like CT, MRI, and PET scans, could further increase diagnostic precision and reliability.

## Discussion and limitations

### Clinical importance and practical relevance

The growing use of AI-generated manipulations in medical imaging poses a serious risk to diagnostic accuracy and patient safety. In this context, the AFFETDS framework offers practical value as a **pre-screening tool** that can automatically flag suspicious MRI scans before they reach radiologists. Such an automated safeguard can help reduce diagnostic errors caused by deepfakes, especially in high-workload hospital settings where manual verification is time-consuming and often impractical. Integrating AFFETDS into Picture Archiving and Communication Systems (PACS) or institutional quality-assurance workflows could provide an additional layer of protection against tampered data, ensuring that clinicians are alerted to potentially manipulated scans early in the diagnostic process.

### Scope of the study

This study focuses specifically on tumor manipulations in brain MRI scans. The methodological emphasis is on combining adversarial robustness techniques with multimodal feature fusion to detect subtle appearance changes introduced through synthetic tampering. While the results demonstrate the feasibility and effectiveness of this approach, the findings are primarily relevant to MRI-based tumor detection. Nonetheless, the underlying principles—feature fusion, ensemble prediction, and adversarial training—have the potential to be transferred to other biomedical imaging contexts.

### Limitations of the proposed model

Although the proposed framework demonstrates promising performance, several limitations should be acknowledged for transparency and future improvement: Despite its strong performance, the proposed AFFETDS framework has several limitations that should be acknowledged. This study exclusively is based on brain MRI. Other imaging modalities such as CT, PET, or X-ray exhibit different noise patterns, contrast levels, and structural characteristics, and the capability of AFFETDS to handle these modalities remains unexplored. The manipulated tumor images used for training and testing were generated synthetically through computational techniques, which, although effective in simulating realistic tampering, may not capture the full complexity of real-world adversarial attacks. Finally, due to computational constraints, the model was not benchmarked against the latest transformer-based or state-space architectures, which limits the breadth of comparison with cutting-edge deepfake detection methods.

## Conclusion

The emergence of deepfake technology poses serious challenges for medical imaging. Clear visual data is essential for accurate diagnosis and treatment. To tackle this issue, the proposed Adversarial Feature Fusion Ensemble (AFFETDS) for Tumor Detection in Medical Imaging combines the handcrafted features with deep learning representations. This approach captures efficiently both detailed information and broader patterns. As a result, it improves the system’s ability to spot minor manipulations caused by tumor deepfakes boosting overall accuracy and trustworthiness.

AFFETDS increases the performance by using ensemble classification which merges predictions from multiple classifiers based on how well they perform individually. This approach ensures strength and adaptability across different datasets. Experimental results indicate that AFFETDS surpasses traditional methods like SVM (AUC ≈ 0.75) and CNN (AUC ≈ 0.78), achieving an AUC of about 0.80 in ROC analysis. Additionally, precision rates from 0.75 to 0.85 and recall rates from 0.70 to 0.80 confirm its capability to accurately distinguish real tumor images from synthetic ones.

By merging feature fusion techniques with ensemble learning strategies, AFFETDS solidifies medical image security, which helps protect patient safety and maintain the quality of diagnostic procedures. Its strong performance demonstrates its potential as a vital solution in the ongoing battle against tumor deepfakes in medical imaging.

## Supplementary Information

Below is the link to the electronic supplementary material.

Supplementary Material 1

## Data Availability

The data underlying this study are drawn from trusted, publicly accessible sources:1. The Cancer Imaging Archive (TCIA): Real MRI scans used in this research can be accessed at https://www.cancerimagingarchive.net/. TCIA provides a comprehensive collection of cancer-related imaging data for scientific research.2. Alzheimer’s Disease Neuroimaging Initiative (ADNI): Additional genuine MRI scans are available at https://adni.loni.usc.edu/data-samples/. ADNI is a widely used repository of high-quality neuroimaging and related data.
