# Supplementary material for: Enhancing tumor deepfake detection in MRI scans using adversarial feature fusion ensembles
Source: Sci Rep. 2025 Dec 9;16:1667. doi: 10.1038/s41598-025-31231-7 (PMC12800179; doi:10.1038/s41598-025-31231-7)
Supplement: Supplementary file 1 — Supplementary Material 1 [file 41598_2025_31231_MOESM1_ESM.docx]

**Data Availability Statement**

The data underlying this study are drawn from trusted, publicly accessible sources:

1. The Cancer Imaging Archive (TCIA): Real MRI scans used in this research can be accessed at https://www.cancerimagingarchive.net/. TCIA provides a comprehensive collection of cancer-related imaging data for scientific research.
2. Alzheimer’s Disease Neuroimaging Initiative (ADNI): Additional genuine MRI scans are available at https://adni.loni.usc.edu/data-samples/. ADNI is a widely used repository of high-quality neuroimaging and related data.

Altered (synthetic) MRI images for model evaluation were created as part of this study using detailed protocols described in the supplementary material. These synthetic datasets, as well as the code for generating them, are available from the corresponding author on reasonable request, in accordance with institutional and privacy guidelines.

No patient-identifiable information is included in the datasets. For further questions or data access not covered by the above links, please contact the corresponding author.
